# ROS-responsive carrier-free nanodrugs for three-pronged acute ischemic stroke therapy

**DOI:** 10.1016/j.mtbio.2026.103531

**Published:** 2026-08-04

**Authors:** Wanquan Lin, Chenmin Fan, Guoyu Xia, Ruifei Li, Hongtao Liu, Zhongxiong Fan, Weiling He, Jingwen Li

**Affiliations:** a Department of Gastrointestinal Surgery, Xiang'an Hospital of Xiamen University, School of Medicine, Xiamen University, Xiamen, Fujian, 361000, China; b School of Pharmaceutical Sciences, Institute of Materia Medica, Xinjiang University, Urumqi, 830017, China; c Department of Cardiology, Fujian Medical Center for Cardiovascular Diseases, Fujian Medical University Union Hospital, Fuzhou, Fujian, China; d Department of Neurosurgery, Xiang'an Hospital of Xiamen University, School of Medicine, Xiamen University, Xiamen, Fujian, 361000, China; e Department of Pharmacy, The First Hospital of Hebei Medical University, Shijiazhuang, 050000, China; f The First Hospital of Qiqihar, No. 700 South Pukui Road, Qiqihar, Heilongjiang Province, 161000, China

**Keywords:** Carrier-free nanodrugs, ROS-Responsive, Antioxidant therapy, Acute ischemic stroke

## Abstract

Acute ischemic stroke (AIS) remains a devastating neurological disorder with limited therapeutic options, in which ischemia/reperfusion-induced oxidative stress, hypoxia, impaired cerebral perfusion, and neuronal injury jointly contribute to disease progression. Here, we report a reactive oxygen species (ROS)-responsive carrier-free nanodrug constructed through microwave-assisted coordination assembly of ruthenium (Ru), L-arginine (L-Arg), and curcumin (Cur), termed RAC. Unlike a simple physical mixture of the three components, RAC represents a coordination-driven carrier-free nanoassembly that integrates multiple therapeutic functions within a single platform, including Ru-associated H_2_O_2_-responsive oxygen generation, L-Arg-associated nitrite/NO-related signaling, and Cur-mediated antioxidant neuroprotection. RAC formed a distinct supramolecular nanostructure and remained relatively stable under physiological conditions, while undergoing ROS/acidosis-responsive disassembly and Cur release in ischemia/reperfusion-relevant microenvironments. *In vitro*, RAC exhibited broad ROS-scavenging capability, improved Cur-associated cellular delivery, reduced intracellular ROS and superoxide accumulation, preserved mitochondrial membrane potential, restored redox homeostasis, and attenuated apoptosis in N2a neuronal cells under OGD/R conditions more effectively than the corresponding physical mixture. *In vivo* fluorescence imaging showed enhanced brain accumulation of RAC after systemic administration. In a tMCAO/R mouse model, RAC promoted cerebral blood flow recovery, reduced infarct volume, preserved cortical neuronal integrity, and improved neurological outcomes compared with both the model and Mix groups. These findings demonstrate that the coordination-driven nanoassembly of RAC contributes to assembly-dependent therapeutic amplification beyond a simple combination of its individual components. This study provides a promising strategy for AIS therapy through the rational design of ROS-responsive, carrier-free nanodrugs integrating free radical scavenging, oxygen generation, NO-related perfusion support, and neuroprotection.

## Graphical abstract

**Figure:**
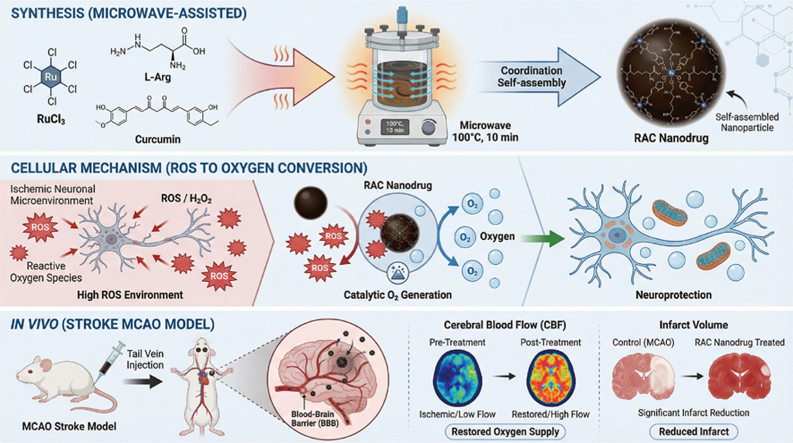

## 1 Introduction

Acute ischemic stroke (AIS) remains a leading cause of mortality and long-term disability worldwide, affecting over 15 million people annually and imposing substantial socioeconomic burden on healthcare systems [1]. Arterial occlusion initiates oxygen-glucose deprivation and a damaging cascade involving excitotoxicity, oxidative stress, inflammation, and neuronal death [2,3]. Current therapies, such as intravenous thrombolysis with recombinant tissue plasminogen activator (rt-PA) and mechanical thrombectomy, are constrained by narrow time windows and hemorrhagic risk, and consequently less than 10% of patients receive timely treatment [[4], [5], [6]]. Moreover, even after successful recanalization, ischemia-reperfusion injury can drive secondary damage that limits penumbral salvage and functional recovery [7]. Therefore, innovative neuroprotective strategies that operate beyond conventional time windows by mitigating ischemia-reperfusion-associated secondary injury are urgently needed.

Among ischemia-reperfusion-associated secondary injury mechanisms, oxidative stress is a key driver of neuronal damage. Of the reactive oxygen species (ROS), hydroxyl radicals (•OH) are particularly deleterious due to their extreme reactivity and short half-life [8]. They cause indiscriminate oxidation of lipids, proteins, and nucleic acids within the ischemic penumbra, which is the potentially salvageable tissue surrounding the infarct core that is highly vulnerable to ROS-mediated injury [9]. Given the brain's high oxygen demand and relatively limited endogenous antioxidant defenses [10], mitigating oxidative injury is therefore essential to preserve penumbral viability and restrain infarct expansion. These pathophysiological features also create a lesion-specific microenvironment that can be harnessed for targeted drug delivery. During ischemia-reperfusion injury, ROS are markedly elevated in the ischemic region, tissue acidosis develops (pH 5.5-6.5), and the blood–brain barrier (BBB) becomes compromised as oxidative injury disrupts endothelial tight junctions [11]. Nanodrug delivery systems (NDDS) have leveraged these cues to improve BBB crossing, prolong systemic exposure, and enhance accumulation in ischemic tissue [12,13].

However, conventional nanocarrier-based NDDS remain difficult to translate for AIS because they often require substantial inert excipients, raise carrier-associated safety concerns, and involve complex syntheses that hinder quality control and scale-up [14,15]. Moreover, these systems often exhibit limited circulation stability and substantial protein adsorption, and the heterogeneous BBB disruption after stroke further reduces their consistent exposure in the ischemic penumbra within the narrow therapeutic window [16]. To overcome these constraints, recent years have witnessed growing interest in carrier-free, self-assembled nanodrugs composed entirely of therapeutic molecules, which maximize active payload and minimize carrier-related uncertainties [17]. Despite this progress, a key remaining challenge for carrier-free nanodrugs is to maintain blood stability while undergoing lesion-activated disassembly in response to excessive ROS and local acidosis, thereby enabling efficient brain delivery and synergistic neuroprotection against ischemia–reperfusion injury.

**Scheme 1:**
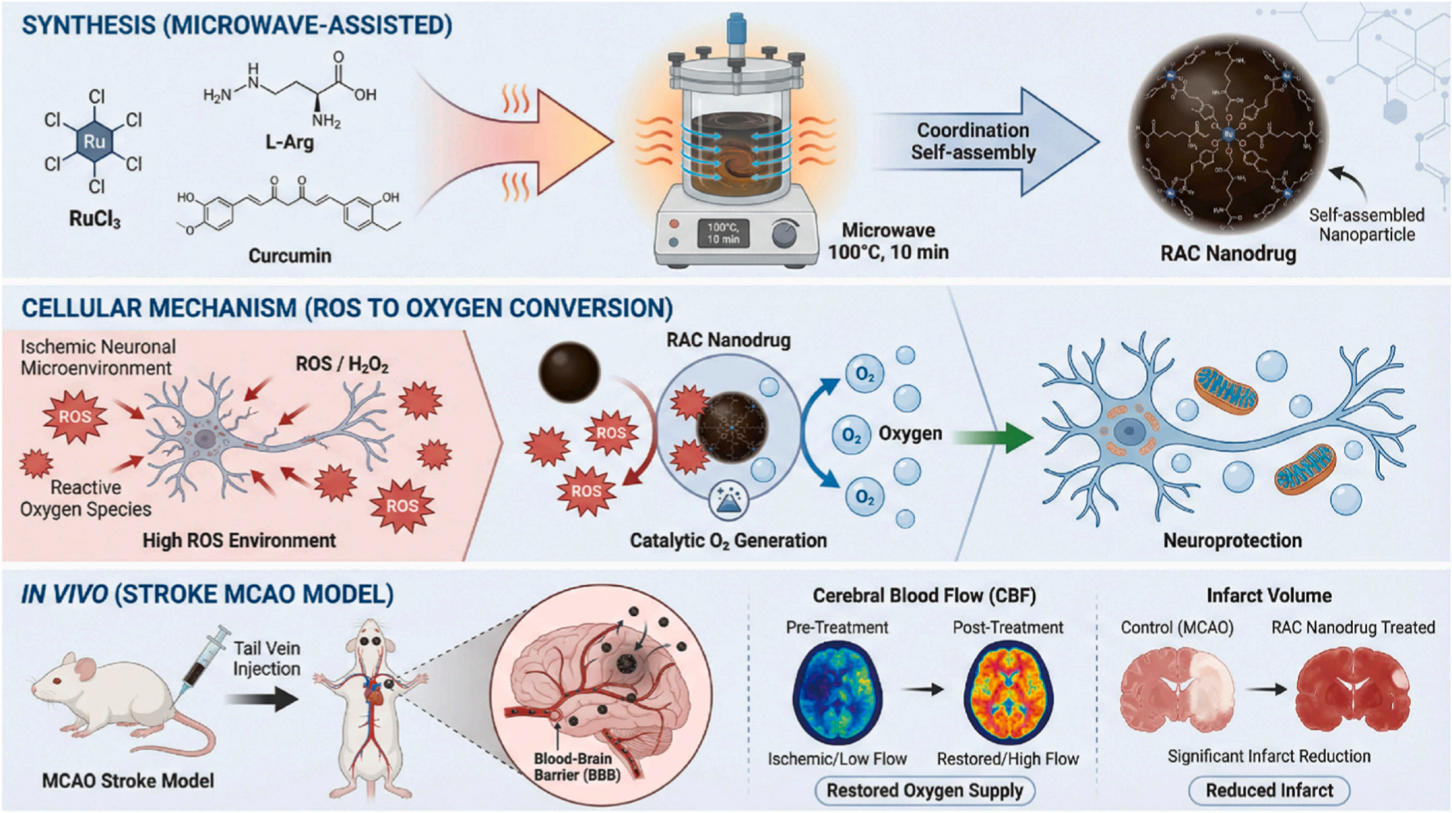
Schematic illustrating the construction of ROS-responsive carrier-free RAC nanodrugs and their therapeutic effects on acute ischemic stroke. Gas generation, nitrite/NO-related signaling and curcumin release from RAC under stimuli of the ROS exert an outstanding synergistic therapeutic effect on acute ischemic stroke by alleviating hypoxia, restoring cerebral blood flow, and attenuating neuronal injury.

Herein, we developed a ROS-responsive carrier-free nanodrug constructed through a microwave-assisted coordination reaction among ruthenium (Ru), L-arginine (L-Arg), and curcumin (Cur), termed RAC (Scheme 1). In RAC, Ru provides catalase-mimicking activity to decompose pathological hydrogen peroxide (H_2_O_2_) and relieve hypoxia, L-Arg serves as a nitric oxide precursor to promote vasodilation and improve cerebral perfusion, and Cur contributes broad antioxidant neuroprotection but is otherwise limited by extremely poor aqueous solubility and low bioavailability. Co-assembling these therapeutics into a single carrier-free nanoplatform increases effective payload while enabling lesion-relevant activation in the ROS-rich, mildly acidic ischemic microenvironment.

In this study, we characterize RAC and evaluate its therapeutic potential for AIS. To further determine whether the therapeutic benefits of RAC arise from assembly-dependent synergy rather than simple component addition, a physical mixture of RuCl_3_, L-Arg, and Cur prepared at the same feeding mass ratio used for RAC assembly (RuCl_3_:L-Arg:Cur = 3:2:1), but without microwave-assisted coordination assembly, was included as a control throughout the *in vitro* and *in vivo* experiments and designated Mix. Although existing AIS nanotherapeutic strategies have incorporated antioxidant, hypoxia-relieving, or ROS-responsive components, many such systems still rely on inert carriers, suffer from limited drug loading, or involve complex multi-component architectures that hinder clinical translation. The carrier-free design of RAC circumvents these limitations by enabling the active therapeutic agents themselves to serve as structural building blocks. Ruthenium was selected for its well-documented catalase-like and H_2_O_2_-responsive catalytic activity, as well as its ability to participate in coordination interactions with the amino and carboxyl groups of L-Arg and the oxygen-containing moieties of Cur, thereby integrating catalytic function and structural assembly within a single platform. The resulting RAC nanoassembly is not merely a physical combination of the three active components but an assembly-amplified therapeutic system in which coordination-driven nanostructure formation enhances cellular uptake, brain accumulation, and ROS-responsive synchronized action.

## 2 Results and discussion

### 2.1 Construction and characterization of nanodrugs

**Fig. 1:**
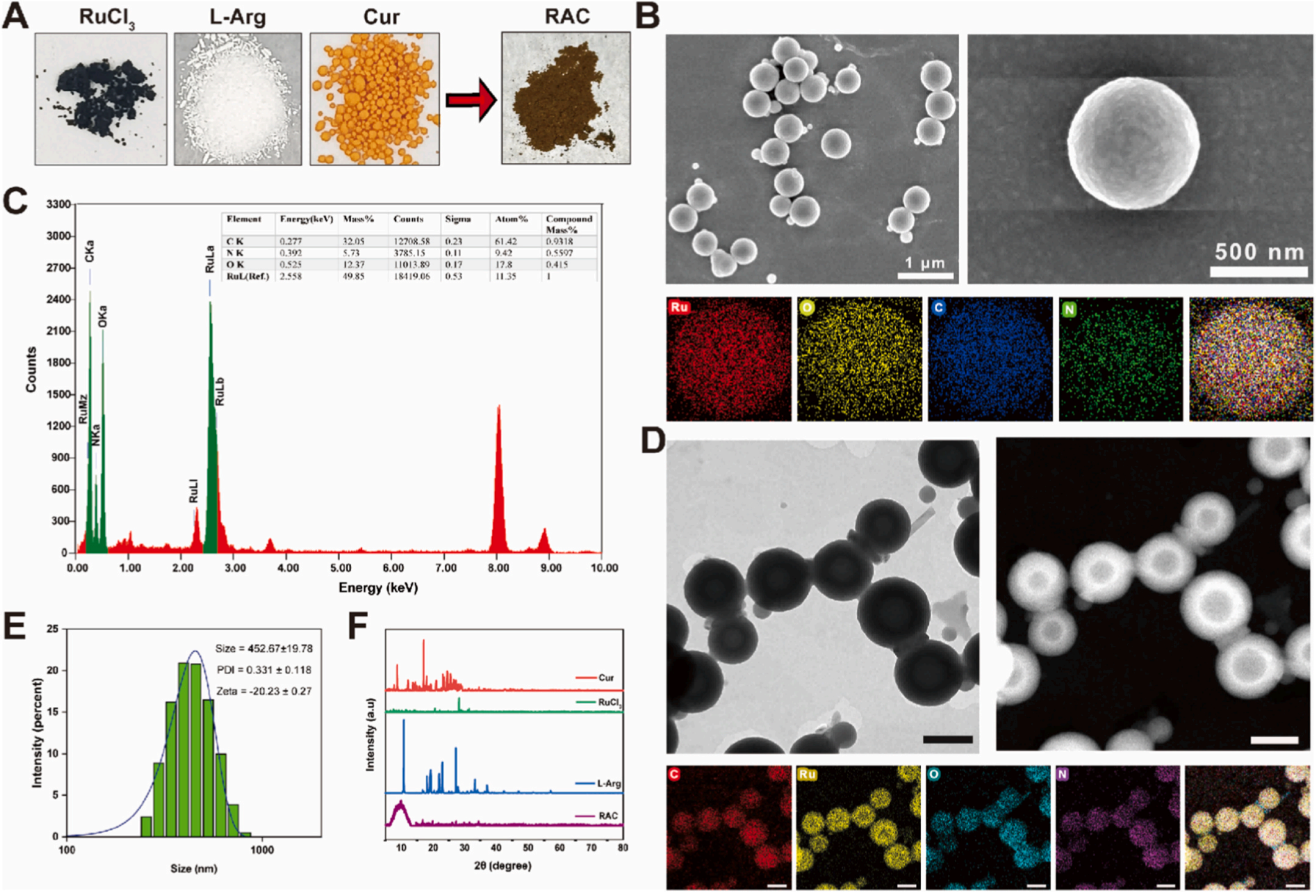
Synthesis and characterization of RAC. (A) A novel RAC carrier-free nanodrug was synthesized from RuCl_3_, L-Arg and Cur. (B) Morphological analysis of RAC by scanning electron microscopy. (C) EDS elemental mapping of RAC, showing the distribution of C, N, O, and Ru. (D) TEM images showing the homogeneous dispersion of electron-dense Ru clusters within the organic matrix. (E) Size distribution and zeta potential. (F) X-ray diffraction analysis.

The rational design of carrier-free nanodrugs requires careful consideration of both molecular interactions and responsiveness to pathological microenvironments [18]. We employed microwave-assisted synthesis to construct RAC nanodrugs through coordination-driven self-assembly of RuCl_3_·xH_2_O, L-Arg, and Cur at a mass ratio of 3:2:1 (Fig. 1A). Unlike conventional physical mixing, microwave irradiation provides rapid and uniform heating, facilitating coordination bond formation between Ru ions and the carboxyl/amino groups of L-Arg, while simultaneously enabling hydrogen bonding interactions with Cur's phenolic hydroxyl and carbonyl groups [19]. This synthetic approach avoids the use of external carriers or additional catalysts and enables rapid formation of homogeneous nanostructures.

Morphological characterization by scanning electron microscopy (SEM) revealed that RAC nanodrugs exhibited a uniform spherical architecture with smooth surfaces (Fig. 1B), indicating successful self-assembly without aggregation. Energy-dispersive X-ray spectroscopy (EDS) mapping confirmed the uniform distribution of key elements, including carbon (C), nitrogen (N), oxygen (O), and Ru, throughout the nanostructures, which validated the chemical composition of the assembled supramolecular systems (Fig. 1C). Transmission electron microscopy (TEM) further showed that electron-dense Ru clusters were homogeneously dispersed within the lighter organic matrix composed of L-Arg and Cur (Fig. 1D). This homogeneous distribution is functionally advantageous: it ensures that Ru catalytic sites are fully accessible for ROS-associated reactions, while the organic components stabilize the nanostructure under physiological conditions and enable disassembly in response to pathological cues.

Dynamic light scattering (DLS) analysis demonstrated that RAC nanodrugs possessed a hydrodynamic diameter of 452.67 ± 19.78 nm with a polydispersity index (PDI) of 0.331 ± 0.118, suggesting a moderately dispersed size distribution (Fig. 1E). The zeta potential measurement yielded a slightly negative surface charge, which may reduce nonspecific protein adsorption and support colloidal stability in biological fluids. Although RAC exhibited a relatively large hydrodynamic diameter compared with many conventional small-sized nanocarriers, hydrodynamic size alone does not fully determine the biological transport behavior of coordination-driven nanoassemblies. Thus, the DLS data were used to define the colloidal characteristics of RAC, whereas its endothelial barrier transport and brain accumulation were assessed separately in subsequent biological experiments.

Colloidal stability was further evaluated by monitoring the hydrodynamic diameter of RAC in DMEM supplemented with 10% fetal bovine serum over 7 days. As shown in Fig. S1, RAC maintained a relatively stable particle size throughout the incubation period, indicating favorable colloidal robustness under physiologically relevant conditions.

X-ray diffraction (XRD) analysis provided strong evidence that RAC nanodrugs are not a simple physical mixture of the three components, but instead form a distinct assembled phase (Fig. 1F). Free Cur exhibited characteristic sharp reflections at 2θ = 10°, 20°, and a series of peaks between 20° and 30°, consistent with its crystalline nature. L-Arg likewise showed pronounced peaks at 2θ = 10° together with multiple reflections between 20° and 30° region, whereas RuCl_3_ showed a broad, low-intensity profile with a weak feature near 30°, in line with its hydrated, poorly crystalline/amorphous character. In contrast, the RAC nanodrug diffractogram was markedly altered. The characteristic crystalline peaks of Cur and L-Arg were largely suppressed and replaced by a broad amorphous halo centered at 2θ = 10° with significantly reduced intensity. These data indicate a transition from crystalline to predominantly amorphous features, suggesting that coordination interactions and hydrogen bonding disrupt the native crystal lattices of the individual components and supporting the formation of a new supramolecular assembly. Consistently, selected-area electron diffraction (SAED) (Fig. S2) exhibited diffuse rings characteristic of amorphous or poorly crystalline materials, rather than the discrete spots expected for well-crystallized phases.

**Fig. 2:**
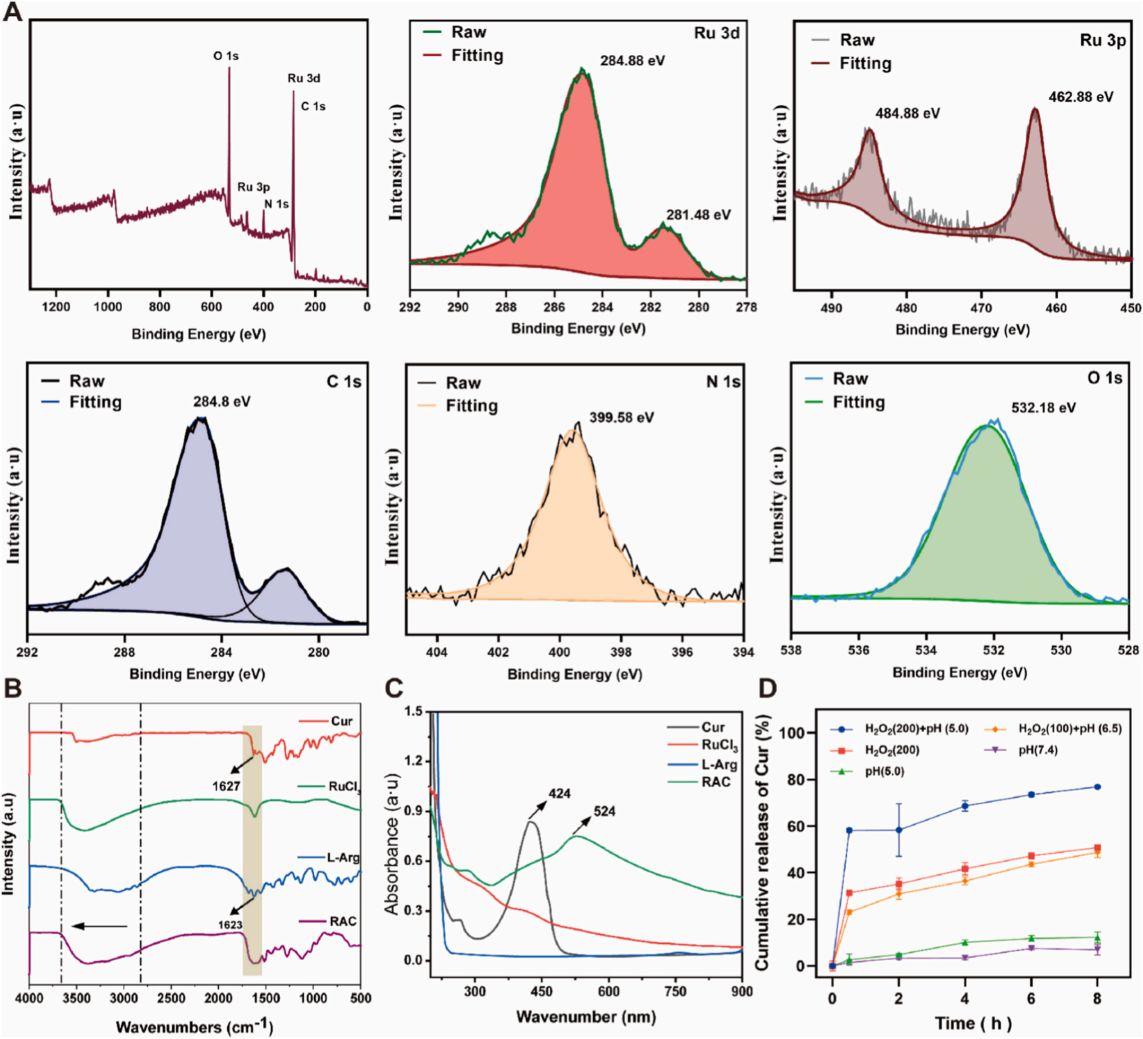
Characterization of RAC. (A) XPS analysis. (B) FT-IR spectra of Cur, RuCl_3_, L-Arg, and RAC. (C) UV-Vis spectra of Cur, RuCl_3_, L-Arg, and RAC. (D) ROS-responsive and acidity-enhanced release profiles.

To elucidate the chemical interactions underlying RAC assembly, we performed X-ray photoelectron spectroscopy (XPS) and Fourier transform infrared spectroscopy (FT-IR) analyses (Fig. 2A and B). High-resolution XPS spectra showed distinct Ru signals, with Ru 3p_1/2_ at 484.88 eV and Ru 3p_3/2_ at 462.88 eV, which are consistent with Ru species in the +3 oxidation state reported in related Ru-ligand systems [20]. The C 1s envelope was deconvoluted into components at 284.88 eV (C-C/C-H), 286.48 eV (C-O/C-N), and 288.78 eV (C=O), indicating carbon in different chemical environments. The N 1s peak at 399.58 eV was assigned to amino groups from L-Arg, and the O 1s peak at 532.18 eV corresponded to oxygen in carboxyl and phenolic functionalities. Notably, modest binding-energy shifts relative to the free components suggest electronic redistribution upon assembly, consistent with Ru–ligand coordination interactions [21]. FT-IR analysis corroborated these observations, as several characteristic bands of the individual components shifted and/or weakened in RAC. Specifically, the phenolic O–H stretching band of Cur (∼3500 cm^−1^) broadened and overlapped with the N-H stretching region of L-Arg, consistent with an extensive hydrogen-bonding network. In addition, the C=O stretching band of Cur at 1627 cm^−1^ and the carboxylate-related vibrations of L-Arg around 1590 cm^−1^ evolved into a single broad band in RAC, suggesting the involvement of these groups in coordination with Ru ions. Collectively, the XPS and FT-IR signatures indicate that RAC formation is driven by multiple noncovalent interactions, including Ru(III) coordination with carboxylate/amino groups and hydrogen bonding among the components, yielding a stable supramolecular architecture.

UV-Vis spectroscopy provided additional insights into the electronic structure of RAC (Fig. 2C). Free Cur showed an absorption maximum at 424 nm, attributed to the π-π* transition of its conjugated diketo-enol chromophore. RuCl_3_ showed broad absorption over the visible region, which can be reasonably attributed to d-d transitions together with ligand-to-metal charge-transfer (LMCT) contributions typical of Ru(III) coordination environments [22]. Upon assembly into RAC, the spectrum displayed a red-shifted Cur peak and a broadened absorption profile extending into the near-infrared region. Such bathochromic shifts upon metal-ligand/coordination-driven assembly have been widely observed in metal-coordinated supramolecular nanostructures and are consistent with a modified electronic environment in RAC [23]. This spectral change also supports the use of RAC fluorescence in subsequent cellular-uptake studies. In addition, the spectral profile of RAC differed from that of the physical mixture of the individual components, further supporting that RAC is not a simple mixture but a distinct assembled species.

### 2.2 *In vitro* on-demand drug release

A defining feature of RAC nanodrugs is their ROS-responsive drug release behavior, which was evaluated via *in vitro* dialysis under simulated physiological and pathological conditions (Fig. 2D). To quantify Cur release, a UV–Vis calibration curve of Cur was established and used for concentration determination (Fig. S3). Under normal physiological conditions (pH 7.4, without H_2_O_2_), Cur release from RAC remained minimal (<20%) over 8 h, demonstrating excellent stability that prevents premature drug leakage during circulation. This stability is attributed to the strong coordination and hydrogen-bonding networks that maintain structural integrity. Upon exposure to H_2_O_2_ (200 μM) at pH 7.4 as an oxidative challenge relevant to ischemia-reperfusion injury, cumulative Cur release increased to ∼50% within 8 h. The ROS-triggered release is attributed to disruption of the coordination-stabilized assembly under oxidative conditions, which is consistent with the use of ROS-responsive nanodrugs in ischemic stroke and related pathologies [24]. To further approximate a milder ischemia/reperfusion-relevant microenvironment, Cur release was also evaluated under pH 6.5 with 100 μM H_2_O_2_. This condition promoted greater Cur release than physiological pH 7.4 without H_2_O_2_, but remained lower than the more stringent pH 5.0 plus 200 μM H_2_O_2_ condition, supporting a graded ROS/acidosis-responsive release behavior of RAC. Notably, acidic pH alone (pH 5.0, without H_2_O_2_) did not substantially promote Cur release, remaining low throughout the test period, indicating that protonation is insufficient to drive disassembly by itself. In sharp contrast, combining H_2_O_2_ with pH 5.0 produced the fastest and highest release, reaching ∼75% within 8 h, highlighting a clear ROS–acidosis synergistic effect. The enhanced release under acidic conditions likely arises from protonation of carboxylate groups, which weakens metal-ligand coordination and destabilizes the assembly; analogous acidosis-responsive nanodrugs have been explored for ischemic stroke management [25,26]. Overall, this dual-responsive behavior, dominated by ROS and enhanced by acidity, enables spatiotemporally controlled delivery, with RAC remaining relatively stable under physiological conditions while undergoing accelerated disassembly in oxidative and acidic microenvironments characteristic of ischemic injury.

In summary, the construction of RAC nanodrugs exemplifies a rational nanodrug design strategy, in which microwave-assisted self-assembly yields a supramolecular architecture stabilized by coordination interactions and hydrogen bonding. The resulting carrier-free nanostructure displays an amorphous profile distinct from its crystalline precursors and dual ROS/pH-responsive release kinetics. Collectively, these attributes support RAC as a potential platform for stroke-targeted therapy by enhancing accumulation in ischemic brain tissue and enabling microenvironment-triggered drug release in lesions.

### 2.3 *In vitro* antioxidant capacity

The pathological cascade of ischemic stroke is strongly associated with oxidative stress. Excessive production of ROS, including H_2_O_2_, superoxide (O2^-^•) and •OH, drives lipid peroxidation, protein oxidation, and DNA damage, thereby triggering neuronal death pathways such as apoptosis [27]. The brain's unique vulnerability to oxidative stress stems from its high metabolic rate, abundant polyunsaturated fatty acids, relatively low antioxidant enzyme expression, and elevated transition metal content, collectively rendering neural tissue exquisitely sensitive to ROS-mediated injury [28]. Given RAC's design as a ROS-responsive nanotherapeutic integrating Ru's catalytic activity, L-Arg's NO precursor function, and Cur's broad antioxidant properties, we conducted comprehensive *in vitro* antioxidant profiling under conditions mimicking the oxidative burst of ischemia-reperfusion injury.

#### 2.3.1 Hydroxyl radical scavenging

**Fig. 3:**
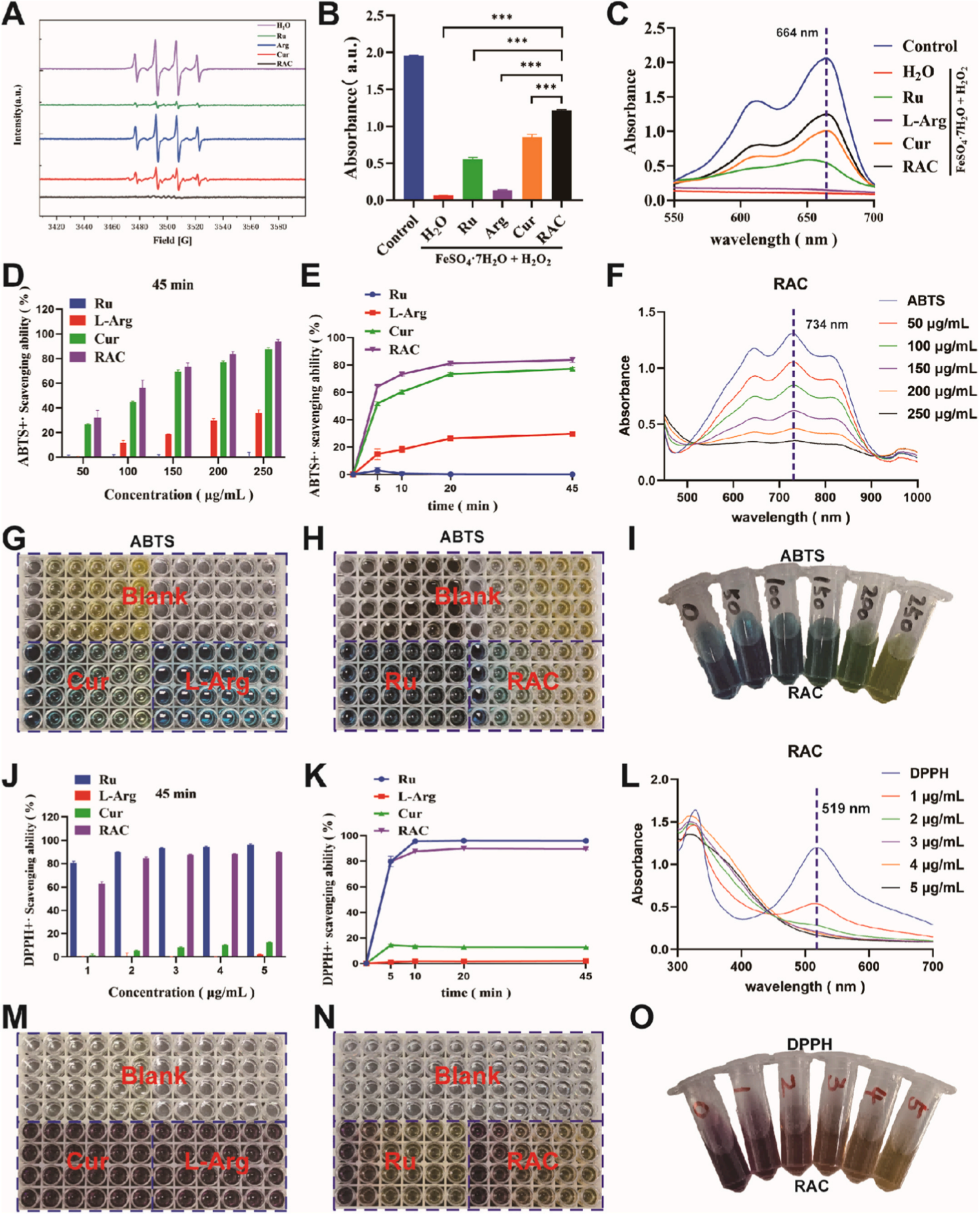
Comprehensive *in vitro* antioxidant profiling and radical scavenging capacity of RAC. (A) EPR spectra of •OH generated in a Fenton reaction system and detected using DMPO as a spin-trapping agent. (B, C) Evaluation of •OH scavenging activity through MB degradation assays. The UV-Vis absorption spectra and corresponding quantitative absorbance at 664 nm demonstrate that higher absorbance values correlate with reduced MB degradation and superior radical scavenging performance of RAC compared with other components. (D, E) Concentration-dependent and time-dependent ABTS+• radical scavenging efficiency of RuCl_3_, L-Arg, Cur, and RAC. The time-dependent kinetics were measured at a fixed concentration of 200 μg/mL (F, I) UV-Vis absorption spectra and corresponding photographs of ABTS+• radicals treated with RAC at various concentrations (0-250 μg/mL), showing the characteristic bleaching at 734 nm. (G, H) Representative microplate images displaying the visual colorimetric changes during the ABTS+• scavenging assay for different treatment groups. (J, K) Concentration-dependent and time-dependent DPPH• radical scavenging activity of the respective components. The kinetic profiles were recorded at a fixed concentration of 5 μg/mL (L, O) UV-Vis absorption spectra and photographs of DPPH• radicals treated with RAC at concentrations from 0 to 5 μg/mL illustrating the concentration-dependent absorbance decrease at 519 nm and the corresponding transition from purple to pale yellow. (M, N) Representative microplate images of the DPPH• radical scavenging assay documenting the antioxidant capacity of each group. Data are presented as mean ± SEM (n = 4). Statistical significance is indicated by *P < 0.05, **P < 0.01, and ***P < 0.001. (For interpretation of the references to color in this figure legend, the reader is referred to the Web version of this article.)

•OH, the most cytotoxic ROS species generated during ischemia-reperfusion [29], were quantitatively assessed using Fenton reaction systems combined with 5,5-dimethyl-1-pyrroline N-oxide (DMPO) spin trapping. Electron paramagnetic resonance (EPR) spectroscopy revealed the characteristic 1:2:2:1 quartet signal of DMPO-OH adducts in the control group (Fig. 3A). Comparative assessment of individual components shows that Cur displayed moderate •OH quenching capacity and Ru exhibited substantial scavenging capacity. Notably, RAC nanodrugs demonstrated the most pronounced suppression of the DMPO-OH signal, with peak intensity reduced by approximately 90% compared to the H_2_O group containing the Fenton reaction system, indicating synergistic enhancement.

To further substantiate these findings, the methylene blue (MB) degradation assay provided complementary colorimetric evidence of hydroxyl radical scavenging. MB undergoes irreversible oxidative degradation by •OH, resulting in the loss of its characteristic absorbance at 664 nm [30]. Quantitative spectrophotometric analysis revealed that MB absorbance decreased dramatically in the H_2_O group containing the Fenton reaction system (Fig. 3B). In contrast, RAC afforded the highest protective effect. Full UV-Vis spectra confirmed the preservation of the MB profile in RAC samples (Fig. 3C), and visual inspection showed persistent blue coloration in RAC-containing tubes (Fig. S4). This superior performance suggests that RAC's nanostructure effectively enhances the accessibility of reactive sites to radicals.

These observations can be attributed to the synergistic interactions among the constituent components within RAC's nanostructure. Specifically, Ru undergoes reversible redox cycling (Ru^3+^/Ru^2+^) to catalytically modulate •OH levels, while Cur concurrently donates electrons via its enolic hydrogen to form resonance-stabilized radicals [31,32]. This integrated design not only optimizes the catalytic efficiency of the metal center but also overcomes Cur's inherent pharmaceutical limitations, such as poor aqueous solubility and rapid degradation. By organizing these active species within a nanoarchitecture, RAC preserves their bioactivity and ensures sustained radical neutralization, resulting in the superior antioxidant performance observed in the EPR and MB assays.

#### 2.3.2 ABTS+• radical scavenging activity

The 2,2′-Azino-bis (3-ethylbenzothiazoline-6-sulfonic acid) diammonium salt (ABTS+•) assay evaluates hydrophilic antioxidant capacity by measuring the decolorization of the blue-green radical (*λ*_max_ = 734 nm) [33]. Concentration-dependent studies demonstrated that RAC exhibited dose-dependent scavenging activity across the concentration range of 50–250 μg/mL, achieving nearly 100% elimination at 250 μg/mL (Fig. 3D). Comparative analysis revealed that Cur was the primary contributor to ABTS+• scavenging among the individual components, consistent with its phenolic hydroxyl groups' capacity for single-electron transfer (SET) and hydrogen atom transfer (HAT) mechanisms [34]. RuCl_3_ exhibited modest activity, while L-Arg showed no appreciable ABTS+• scavenging. Notably, RAC consistently outperformed free Cur at equivalent concentrations, suggesting that the nanoformulation enhances antioxidant efficiency potentially through improved dispersion and synergistic metal-ligand interactions.

Time-course experiments revealed rapid ABTS+• scavenging kinetics by RAC nanodrugs, with more than 80% radical elimination achieved within 20 min and maximal scavenging maintained thereafter (Fig. 3E). This rapid response is critical for therapeutic efficacy because the oxidative burst during reperfusion occurs within minutes to hours following recanalization [35]. UV-Vis spectral analysis confirmed progressive bleaching of the ABTS+• absorption band at 734 nm with increasing RAC concentrations (Fig. 3F), corroborated by the visual color transition from deep green (ABTS+•) to colorless (reduced ABTS) in RAC samples (Fig. 3G-I).

#### 2.3.3 DPPH• radical scavenging activity

The 2,2-Diphenyl-1-picrylhydrazyl (DPPH•) assay complements the ABTS system by evaluating lipophilic antioxidant activity, which is relevant for protecting lipid-rich neuronal membranes from peroxidation. DPPH• is a stable radical with a deep purple coloration (*λ*_max_ = 519 nm) that turns pale yellow upon reduction by hydrogen-donating antioxidants [36]. Assays revealed that RuCl_3_ was the most potent individual scavenger, achieving more than 90% elimination at 5 μg/mL (Fig. 3J). In comparison, RAC nanodrugs also demonstrated robust DPPH• scavenging, though slightly attenuated relative to pure RuCl_3_ due to the dilution of Ru content within the nanoassembly. Conversely, Cur showed minimal activity, and L-Arg was inactive.

Temporal analysis demonstrated that DPPH• scavenging by both RuCl_3_ and RAC reached maximum efficiency within 20 min (Fig. 3K), consistent with the rapid kinetics observed for ABTS+•. To further characterize the scavenging process spectroscopically, monitoring (400–700 nm) was performed on RAC samples, which documented the characteristic loss of DPPH• absorbance at 519 nm (Fig. 3L). The corresponding visual color change from purple to yellow provided independent qualitative confirmation of the quantitative spectroscopic data (Fig. 3M-O).

In summary, comprehensive *in vitro* antioxidant profiling demonstrates that RAC nanodrugs possess exceptional, broad-spectrum scavenging capabilities against hydroxyl radicals, ABTS+•, and DPPH•. This performance arises from the synergistic integration of Cur's phenolic antioxidant properties with Ru's catalytic redox activity within a stable nanoarchitecture. Crucially, these findings establish that RAC possesses a dual capacity to neutralize both hydrophilic and lipophilic radicals, positioning it as a versatile antioxidant capable of protecting diverse subcellular compartments, including the aqueous cytosol and lipid bilayers. This multifaceted ROS-neutralizing capacity, combined with rapid scavenging kinetics, represents a critical attribute for comprehensive neuroprotection in AIS. Collectively, these attributes position RAC as a promising therapeutic agent for combating oxidative stress-mediated neuronal injury, with direct implications for preserving tissue viability in the ischemic penumbra and improving functional outcomes.

### 2.4 *In vitro* cytotoxicity and cellular internalization kinetics

Prior to evaluating therapeutic efficacy, a rigorous assessment of the cytocompatibility and cellular uptake kinetics of RAC is essential to establish its safety profile and delivery efficiency. N2a cells, a murine neuroblastoma cell line previously used in oxygen-glucose deprivation/reoxygenation models to investigate ischemia/reperfusion-like neuronal injury, were employed as the cellular model [37].

#### 2.4.1 Cytocompatibility evaluation

Cytotoxicity assays revealed distinct, dose-dependent safety profiles among the various components. While individual components, RuCl_3_ and L-Arg, exhibited negligible cytotoxicity (cell viability >80%) within the tested concentrations, both Cur and RAC nanodrugs displayed concentration-dependent toxicity (Fig. S5A and B). Notably, RAC showed slightly higher cytotoxicity than free Cur at equivalent concentrations, which may be attributed to the significantly enhanced cellular internalization and the synergistic bioactivity of its multiple therapeutic constituents.

#### 2.4.2 Cellular internalization kinetics

To further verify whether RAC could improve intracellular delivery, the cellular internalization kinetics of RAC and free Cur were evaluated in N2a cells by flow cytometry using the intrinsic fluorescence of Cur (Fig. S6A and B). Both formulations exhibited time-dependent uptake over 2, 4, 6, and 8 h. Notably, at 2 h, no significant difference in mean fluorescence intensity (MFI) was observed between RAC and free Cur, suggesting comparable initial uptake rates. However, from 4 h onward, RAC displayed significantly higher MFI values than free Cur, and this difference became progressively more pronounced at 6 h and 8 h, indicating that the nanoassembly structure confers a sustained advantage in cellular internalization over time. This sustained divergence in uptake kinetics is consistent with the assembly-dependent delivery properties of RAC. Unlike free Cur, which is limited by poor aqueous dispersibility, RAC formed a distinct supramolecular nanostructure rather than a simple physical mixture, as supported by XRD, SAED, XPS, FT-IR, and UV-Vis analyses. In addition, RAC exhibited a nanoscale hydrodynamic size, a mildly negative surface charge, and favorable colloidal stability in serum-containing medium. These physicochemical features may maintain RAC in a dispersed and structurally stabilized nanoassembled state, thereby increasing the effective availability of Cur-containing assemblies for cell-membrane contact and sustained intracellular accumulation. Therefore, the enhanced MFI observed after 4 h is more likely attributable to nanoassembly-mediated improvement in Cur dispersion, stability, and cellular delivery.

### 2.5 *In vitro* endothelial barrier transport of RAC

**Fig. 4:**
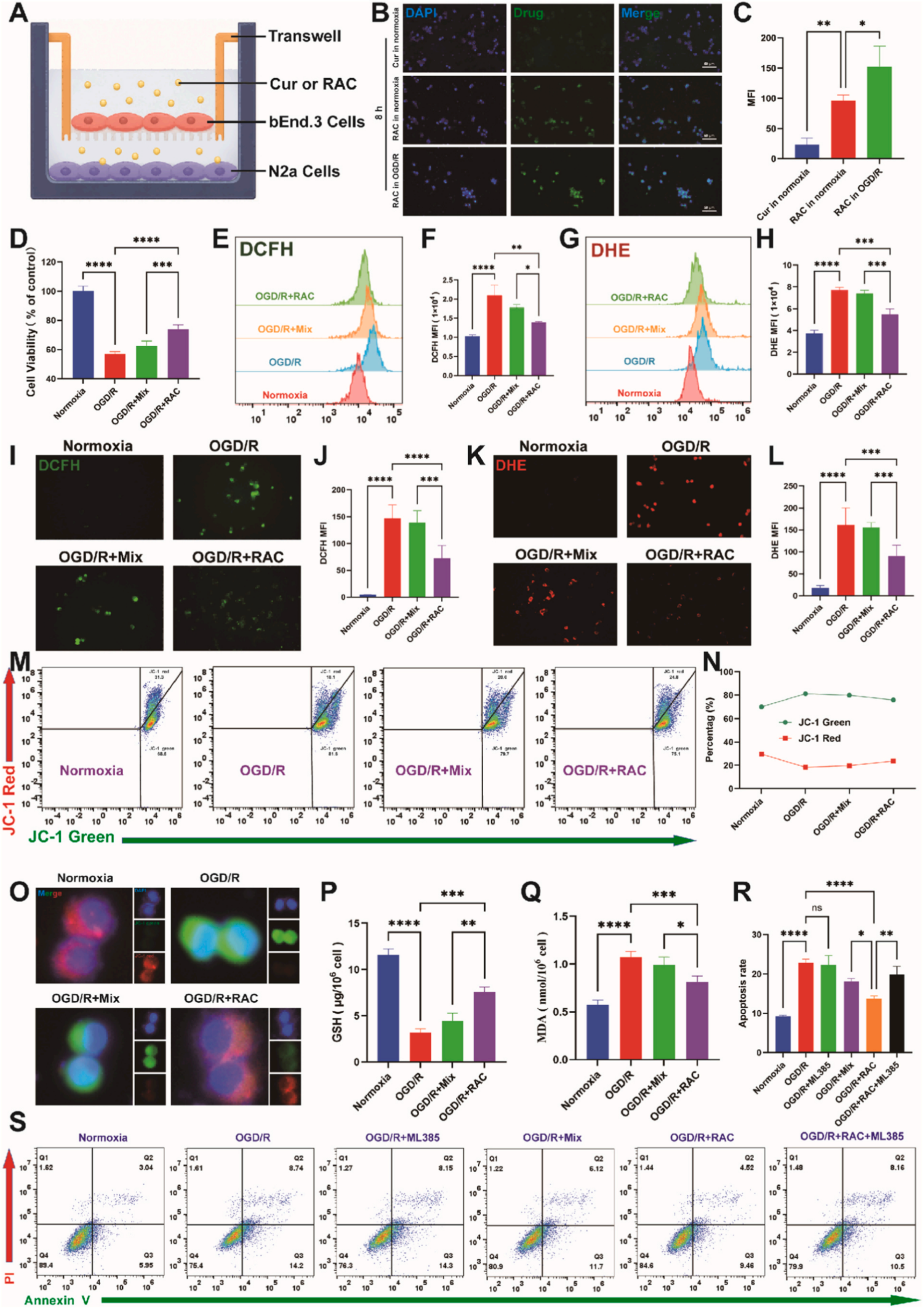
*In vitro* transendothelial transport and neuroprotective effects of RAC. (A) Schematic illustration of the bEnd.3 Transwell endothelial barrier model used to evaluate transendothelial transport, with N2a cells seeded in the lower chamber as recipient cells for fluorescence detection. (B) Representative CLSM images of N2a cells in the lower chamber after treatment with free Cur under normoxia, RAC under normoxia, or RAC under OGD/R-mimicking cellular conditions. (C) Quantitative analysis of fluorescence intensity in the lower chamber under the three tested conditions. (D) Cell viability of N2a cells. (E, F) DCFH-DA-based flow cytometric detection and quantitative analysis of intracellular ROS generation. (G, H) DHE-based flow cytometric detection and quantitative analysis of superoxide production. (I, J) Representative confocal images and fluorescence quantification of DCFH-DA staining. (K, L) Representative confocal images and fluorescence quantification of DHE staining. (M, N) Flow-cytometric analysis of JC-1 red and green fluorescence distributions/percentages in N2a cells after different treatments. (O) Representative JC-1 confocal images. (P) Intracellular GSH content. (Q) MDA level reflecting lipid peroxidation. (R) Quantitative analysis of apoptotic cell proportions. (S) Representative flow cytometric plots of Annexin V/PI apoptosis assay. Data are presented as the means ± SEM. *P < 0.05, **P < 0.01, ***P < 0.001, ****P < 0.0001. (For interpretation of the references to color in this figure legend, the reader is referred to the Web version of this article.)

To evaluate the transendothelial transport capacity of RAC, a bEnd.3 Transwell endothelial barrier model was established, with N2a cells seeded in the lower chamber as recipient cells for fluorescence detection (Fig. 4A). Under normoxic conditions, free Cur and RAC were compared at equivalent Cur concentrations, whereas RAC was further evaluated under OGD/R-mimicking cellular conditions. Compared with free Cur in normoxic cells, RAC produced stronger fluorescence signals in the lower chamber, indicating improved transport across the bEnd.3 endothelial monolayer. Moreover, OGD/R-mimicking cellular conditions further increased RAC-associated fluorescence in the lower chamber, suggesting that endothelial barrier disruption under OGD/R-like cellular stress facilitated RAC transendothelial transport (Fig. 4B and C). These results demonstrate that RAC exhibits enhanced transendothelial transport relative to free Cur, and that OGD/R-mimicking conditions further increase the transport efficiency of RAC across the bEnd.3 endothelial barrier model.

### 2.6 RAC improves N2a neuronal cell viability under OGD/R conditions

Building upon the established cellular internalization and biocompatibility profiles, we further evaluated the cytoprotective efficacy of RAC using the oxygen-glucose deprivation/reperfusion (OGD/R) model in N2a cells. This specific model serves as a robust *in vitro* system to recapitulate the pathological cascades of ischemic stroke, where neuronal cells act as primary responders that are highly susceptible to ischemic insult [37]. Because neuronal injury during reperfusion contributes to secondary brain damage, protecting N2a cells from OGD/R-induced injury provides relevant evidence for direct cytoprotection.

#### 2.6.1 Dose-response optimization

To identify optimal therapeutic dosing, N2a cells were pretreated with a range of RAC concentrations before OGD/R exposure (Fig. S7). Exposure to OGD/R alone significantly compromised cell viability, reducing it to approximately 60% relative to normoxic controls. RAC pre-treatment exhibited a concentration-dependent protective effect at low-to-moderate doses, where cell viability was progressively restored. However, at higher concentrations, a decline in cell viability was observed, likely attributable to the intrinsic cytotoxicity of RAC itself at elevated doses. This dose-response pattern reflects a narrow therapeutic window in which the beneficial cytoprotective effects of RAC must be balanced against its concentration-dependent toxicity, analogous to the classical pharmacological principle that subtherapeutic doses are ineffective while supratherapeutic doses may cause harm. Based on these results, an optimal concentration range was identified for subsequent *in vitro* experiments.

#### 2.6.2 Assembly-dependent synergistic therapeutic efficacy

To further determine whether the enhanced cytoprotection of RAC originated from nanostructure-mediated integration rather than simple co-administration of the three components, a physical mixture (Mix) group was included. The Mix group contained RuCl_3_, L-Arg, and Cur at the same mass ratio as RAC but without microwave-assisted coordination assembly. Under OGD/R conditions, the Mix provided only partial protection, indicating that the simple combination of the three therapeutic moieties was insufficient to fully rescue cell viability. In contrast, RAC significantly restored N2a cell viability to a much higher level than Mix (Fig. 4D). This result demonstrates that the coordinated nanoassembly, rather than a mere additive pharmacological effect, is essential for maximizing the cytoprotective efficacy.

### 2.7 RAC attenuates OGD/R-induced oxidative stress, mitochondrial dysfunction, and lipid peroxidation in N2a cells

Given the broad radical-scavenging capacity of RAC, we next investigated whether RAC could suppress OGD/R-induced oxidative stress in N2a neuronal cells. DCFH-DA-based flow cytometry showed that OGD/R markedly increased intracellular total ROS levels compared with the normoxic control group, whereas Mix partially reduced ROS accumulation and RAC produced a more pronounced inhibitory effect (Fig. 4E and F). Consistently, confocal imaging of DCFH-DA staining showed intense green fluorescence in OGD/R-treated cells, while RAC substantially weakened the fluorescence signal, further confirming its intracellular ROS-scavenging activity (Fig. 4I and J).

Superoxide-associated oxidative stress was further assessed using DHE staining. Flow cytometric analysis showed that OGD/R markedly increased DHE fluorescence intensity compared with the normoxic control group, whereas RAC treatment significantly reduced the DHE signal compared with the OGD/R and Mix groups (Fig. 4G and H). Consistently, confocal imaging revealed strong red fluorescence in OGD/R-treated cells, while RAC treatment visibly weakened this fluorescence signal (Fig. 4K and L). Together with the DCFH-DA results, these findings indicate that RAC attenuates intracellular oxidative stress, including total ROS accumulation and DHE-detectable superoxide-associated signals, under OGD/R-mimicking cellular conditions.

Because mitochondrial dysfunction is a central event in OGD/R-induced neuronal injury, mitochondrial membrane potential was further evaluated using JC-1 staining. JC-1 staining further showed that OGD/R markedly reduced JC-1 red fluorescence and increased JC-1 green fluorescence, indicating mitochondrial depolarization. RAC treatment partially restored the JC-1 red fluorescence distribution and reduced the green fluorescence percentage, suggesting preservation of mitochondrial membrane potential (Fig. 4M and N). Representative confocal images showed the same trend, with RAC preserving stronger red JC-1 aggregate fluorescence and reducing green monomer fluorescence compared with the OGD/R group (Fig. 4O). These findings suggest that RAC helps maintain mitochondrial integrity during OGD/R injury.

Redox homeostasis and lipid peroxidation were further assessed by measuring intracellular GSH and MDA levels. OGD/R depleted intracellular GSH, whereas RAC restored GSH content more effectively than Mix (Fig. 4P). In parallel, OGD/R markedly increased MDA levels, reflecting enhanced lipid peroxidation, while RAC significantly reduced MDA accumulation (Fig. 4Q). Together, these results indicate that RAC protects N2a neuronal cells against OGD/R-induced oxidative injury by suppressing ROS and superoxide accumulation, preserving mitochondrial membrane potential, restoring intracellular antioxidant capacity, and reducing lipid peroxidation.

### 2.8 RAC attenuates OGD/R-induced apoptosis with involvement of Nrf2-associated signaling

Finally, Annexin V/PI staining was performed to determine whether the antioxidant and mitochondrial protective effects of RAC translated into reduced apoptotic cell death. Flow cytometric analysis showed that OGD/R markedly increased the apoptotic rate of N2a cells compared with the normoxic group (Fig. 4R and S). Mix treatment produced only a limited reduction in apoptosis, whereas RAC significantly decreased the proportion of apoptotic cells, consistent with its superior effects on ROS suppression, mitochondrial membrane potential preservation, and lipid peroxidation inhibition. Notably, the anti-apoptotic effect of RAC was partially abolished by the Nrf2 inhibitor ML385, as the OGD/R + RAC + ML385 group showed a higher apoptotic rate than the OGD/R + RAC group. Importantly, ML385 alone did not exacerbate OGD/R-induced apoptosis, as the OGD/R + ML385 group exhibited a comparable apoptotic rate to the OGD/R group, indicating that the reversal of RAC-mediated protection by ML385 was not attributable to ML385-induced cytotoxicity. These findings suggest that Nrf2-related antioxidant signaling contributes, at least in part, to the anti-apoptotic effect of RAC under OGD/R conditions.

### 2.9 Improvement of blood flow reconstruction by RAC in tMCAO/R mice

Beyond direct neuroprotection, the functional restoration of cerebral blood flow represents a critical determinant of clinical outcomes following AIS. Sustained hypoperfusion after arterial recanalization, often termed the no-reflow phenomenon, typically drives secondary neuronal injury through mechanisms involving endothelial swelling and microvascular constriction. To address these pathological barriers, RAC was designed to integrate multiple complementary functions, including oxygen generation, NO-related signaling, and enhanced cerebral delivery, with the aim of improving microvascular reperfusion in ischemic brain tissue.

#### 2.9.1 Validation of ROS-triggered oxygen generation and NO-related nitrite signals

**Fig. 5:**
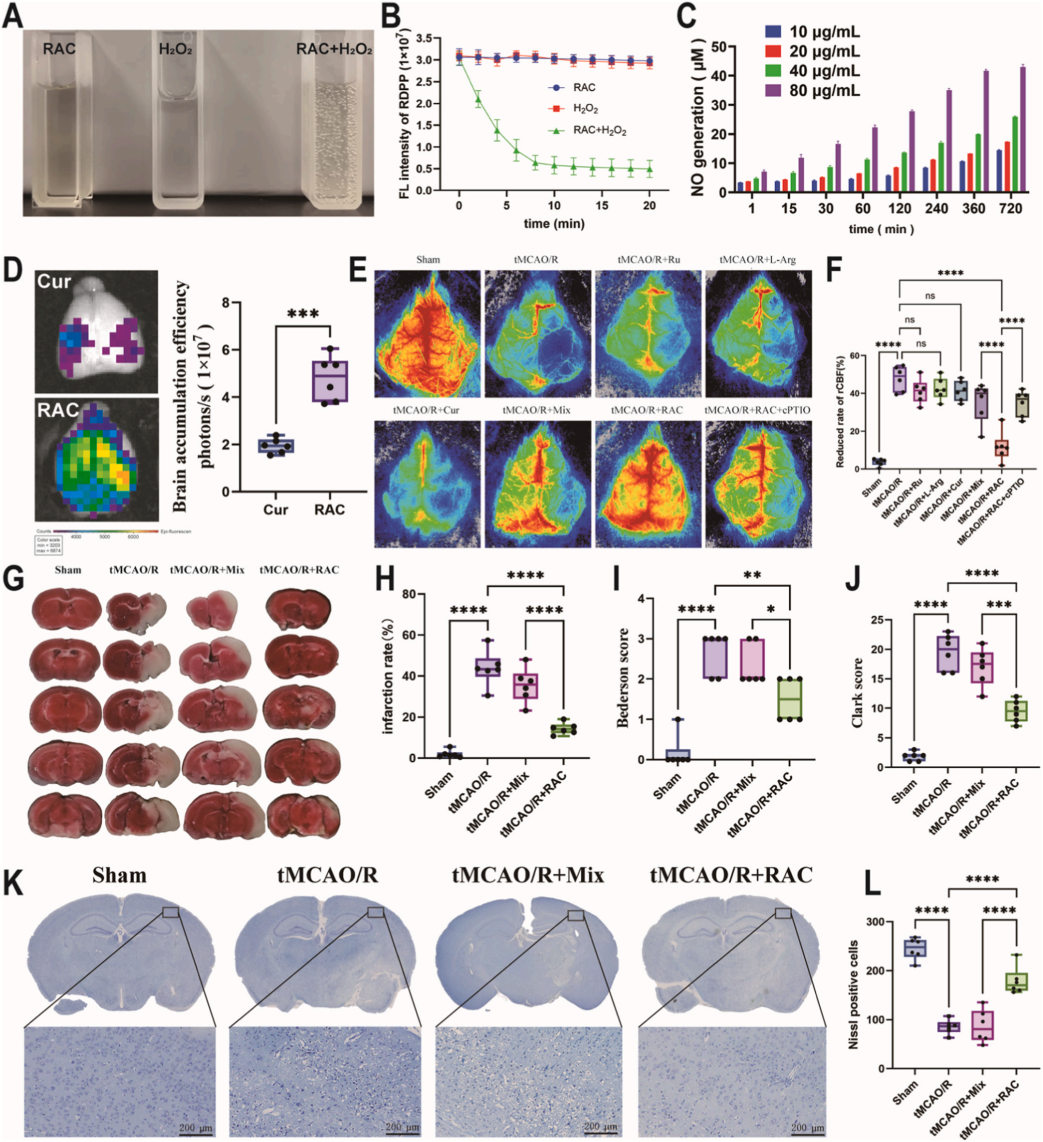
ROS-triggered oxygen generation, NO-related nitrite production, and brain accumulation of RAC, along with its therapeutic efficacy in cerebral blood flow restoration and neuroprotection in tMCAO/R mice. (A) Optical photographs demonstrating catalytic bubble formation in the presence of H_2_O_2_. (B) RDPP fluorescence evidence for ROS-triggered oxygen generation by RAC. (C) Griess reagent detection of nitrite generation (as a surrogate of NO) at different time points and concentrations. (D) Ex vivo fluorescence imaging and quantitative analysis of brain accumulation after intravenous administration of free Cur or RAC. (E) Representative laser speckle contrast images of cortical perfusion in sham, model, Ru, L-Arg, Cur, Mix, RAC, and RAC + cPTIO groups at 24 h post-reperfusion. (F) Quantitative analysis of cerebral perfusion. (G, H) Representative TTC-stained brain sections and quantitative analysis of infarct volume. (I, J) Neurological scores evaluated by Bederson and Clark scoring systems. (K, L) Representative Nissl staining of cortical neurons and quantitative analysis of neuronal density. For box plots, each point represents one animal; boxes indicate median and interquartile range, with whiskers showing min–max. n = 6 per group unless otherwise indicated. Statistical significance is indicated by *P < 0.05, **P < 0.01, ***P < 0.001, ****P < 0.0001.

Before assessing its *in vivo* hemodynamic effects, the ROS-responsive gas-generating behavior of RAC was first examined *in vitro*. Upon exposure to H_2_O_2_, obvious bubble formation was observed in the RAC-containing system (Fig. 5A), indicating that RAC could generate gas under oxidative conditions. Given that Ru-based materials have been widely reported to catalyze the decomposition of H_2_O_2_ into oxygen [[38], [39], [40]], we speculated that the observed gas was mainly oxygen. To verify this assumption, RDPP fluorescence analysis was further performed, as RDPP is an oxygen-sensitive probe whose fluorescence is quenched in the presence of oxygen. RAC or H_2_O_2_ alone caused little change in RDPP fluorescence intensity, whereas the RAC + H_2_O_2_ system induced a rapid and pronounced decrease in RDPP fluorescence over time (Fig. 5B), confirming H_2_O_2_-responsive oxygen generation by RAC. In addition to oxygen generation, the Ru/L-Arg-containing composition of RAC may also support NO-related activity under ROS-rich conditions [[41], [42], [43]]. Therefore, nitrite levels were measured using the Griess assay as a surrogate indicator of NO-related generation. The results showed concentration-dependent and time-dependent increases in nitrite levels after RAC treatment (Fig. 5C). Taken together, these findings indicate that RAC responds to oxidative microenvironments by promoting oxygen generation and NO-related nitrite production, which may contribute to improved cerebral perfusion under ischemic conditions.

#### 2.9.2 Cerebral accumulation capability of RAC

For these ROS-responsive effects to be translated into therapeutic benefits *in vivo*, sufficient brain accumulation is required. Previous studies have shown that brain exposure is closely associated with the solubility, surface properties, and molecular structure of therapeutic agents [44]. Based on this, Cur was used as an intrinsic fluorescent probe to evaluate the brain-associated distribution of RAC by ex vivo fluorescence imaging. As shown in Fig. 5D, RAC produced stronger fluorescence signals in brain tissue than free Cur at 12 h post-injection, and quantitative analysis confirmed enhanced brain-associated fluorescence after RAC administration. This improved exposure is likely attributable to the optimized physicochemical properties of the nanoassembly, which enhance the solubility and stability of Cur during circulation.

#### 2.9.3 Enhancement of cerebral blood flow and microvascular reperfusion

The functional impact of RAC on cerebral hemodynamics was further assessed using laser speckle contrast imaging at 24 h post-reperfusion (Fig. 5E). While sham controls displayed symmetric bilateral cortical perfusion, the tMCAO/R mice exhibited profound perfusion deficits in the right hemisphere, characterized by extensive low-flow regions in cool colors. In the Mix group, cerebral perfusion was partially improved compared with the tMCAO/R group, indicating that co-administration of RuCl_3_, L-Arg, and Cur could provide limited hemodynamic benefit. However, perfusion recovery in the Mix group remained weaker than that observed in the RAC group. RAC treatment led to more evident restoration of cerebral blood flow, as reflected by warmer color distributions in the ischemic hemisphere and a lower reduction rate of relative cerebral blood flow (rCBF). Quantitative analysis further confirmed that RAC outperformed Mix in mitigating perfusion deficits (Fig. 5F). An additional tMCAO/R + RAC + cPTIO group was included in the LSCI experiment to evaluate the contribution of NO-related signaling to RAC-mediated cerebral perfusion improvement, with cPTIO (2 - (4 -carboxyphenyl)- 4,4,5,5 -tetramethylimidazoline- 1- oxyl- 3 -oxide) used as an NO scavenger. cPTIO partially attenuated RAC-mediated cerebral blood flow recovery, suggesting that NO-related signaling contributes to the hemodynamic benefit of RAC. However, the residual protective effect observed in the tMCAO/R + RAC + cPTIO group indicates that oxygen generation, ROS scavenging, and other assembly-dependent effects may also participate in perfusion restoration.

### 2.10 *In vivo* evaluation of infarct volume and neurological function

Given that RAC markedly improved cerebral blood flow after reperfusion, we next examined whether this hemodynamic benefit translated into reduced ischemic injury and improved neurological outcomes in tMCAO/R mice.

#### 2.10.1 Reduction of infarct volume via RAC treatment

To visualize and quantify the extent of cerebral ischemic injury, 2,3,5-triphenyltetrazolium chloride (TTC) staining was performed at 24 h post-reperfusion (Fig. 5G and H). The TTC reagent is specifically reduced by mitochondrial dehydrogenases within metabolically active cells to delineate viable red-stained tissue from necrotic white-stained areas [45]. While the sham controls exhibited uniformly red-stained brain slices which confirmed the absence of ischemic damage, the tMCAO/R mice displayed extensive pale infarction encompassing both the ipsilateral cortex and the striatum. The Mix group showed a moderate reduction in infarct area compared with the tMCAO/R group, suggesting that the combined free components exerted some protective effect. However, this protective effect was still limited, whereas RAC significantly reduced infarct volume compared with Mix and demonstrated superior preservation of metabolically active brain tissue. This difference between RAC and Mix indicates that the therapeutic efficacy of RAC cannot be explained by the simple additive effects of RuCl_3_, L-Arg, and Cur, but instead depends on the formation of the ROS-responsive coordinated nanoassembly.

#### 2.10.2 Amelioration of neurological deficits and functional impairments

Neurological function was further assessed using Bederson and Clark scoring systems at 24 h post-reperfusion (Fig. 5I and J). Sham-operated mice showed no obvious neurological deficits, whereas tMCAO/R mice displayed severe behavioral impairments. Mix treatment partially improved neurological outcomes, but the improvement was limited. In comparison, RAC mice showed significantly lower neurological deficit scores than both the tMCAO/R and Mix groups. These results demonstrate that RAC effectively alleviates neurological dysfunction after ischemic stroke, consistent with its ability to restore cerebral blood flow and reduce infarct volume.

### 2.11 RAC attenuates cortical neuronal damage *in vivo*

Because restoration of cerebral blood flow and reduction of infarct volume are expected to alleviate downstream neuronal injury, we further examined whether RAC could preserve neuronal morphology in the ischemic cortex. Nissl staining was performed on coronal brain sections at 24 h post-reperfusion to evaluate neuronal survival and cortical integrity (Fig. 5K and L). Nissl bodies, which are mainly composed of rough endoplasmic reticulum and ribosomes, are important histological indicators of neuronal viability. Their loss or disruption reflects neuronal injury after ischemia-reperfusion.

In the sham group, cortical neurons exhibited normal morphology, with clear cell bodies, prominent nuclei, and abundant Nissl bodies. In contrast, the tMCAO/R group showed severe neuronal damage, characterized by reduced neuronal density, shrunken cell bodies, pyknotic nuclei, and marked loss of Nissl bodies. The Mix group showed partial preservation of neuronal morphology compared with the tMCAO/R group, indicating that the physical combination of RuCl_3_, L-Arg, and Cur exerted limited neuroprotective effects. However, obvious neuronal loss and cortical disorganization were still observed. By contrast, RAC treatment markedly preserved cortical neuronal structure, as evidenced by more abundant Nissl-positive neurons, higher neuronal density, and more intact tissue architecture. These findings indicate that RAC effectively attenuates neuronal injury and cortical damage after ischemia-reperfusion.

Importantly, the inclusion of the Mix group provides further evidence for the assembly-dependent therapeutic advantage of RAC. Although Mix contained the same therapeutic components at the same ratio, its efficacy in improving cerebral perfusion, reducing infarct volume, preserving neuronal integrity, and improving neurological function was consistently inferior to that of RAC. Together with the superior cytoprotective effect of RAC observed in the OGD/R model, these results indicate that the therapeutic performance of RAC is not simply due to the combination of RuCl_3_, L-Arg, and Cur. Instead, the coordination-driven nanoassembly likely improves aqueous dispersion, stabilizes Cur, enhances cellular uptake and brain accumulation, and enables ROS-responsive synchronized release of multiple active moieties within the ischemic microenvironment. Therefore, RAC represents an assembly-amplified therapeutic system rather than a conventional drug combination.

### 2.12 Biocompatibility and safety assessment of RAC

A rigorous evaluation of biocompatibility, *in vivo* tolerability, and histological safety is a prerequisite for the further development of therapeutic nanomaterials [46]. In this study, the safety and tolerability profile of RAC was evaluated through *in vitro* hemolysis assays, dynamic body weight monitoring, Kaplan-Meier survival analysis, and H&E histopathological examination of major organs and brain tissues.

#### 2.12.1 Mitigation of erythrocyte membrane disruption via nanoassembly formation

**Fig. 6:**
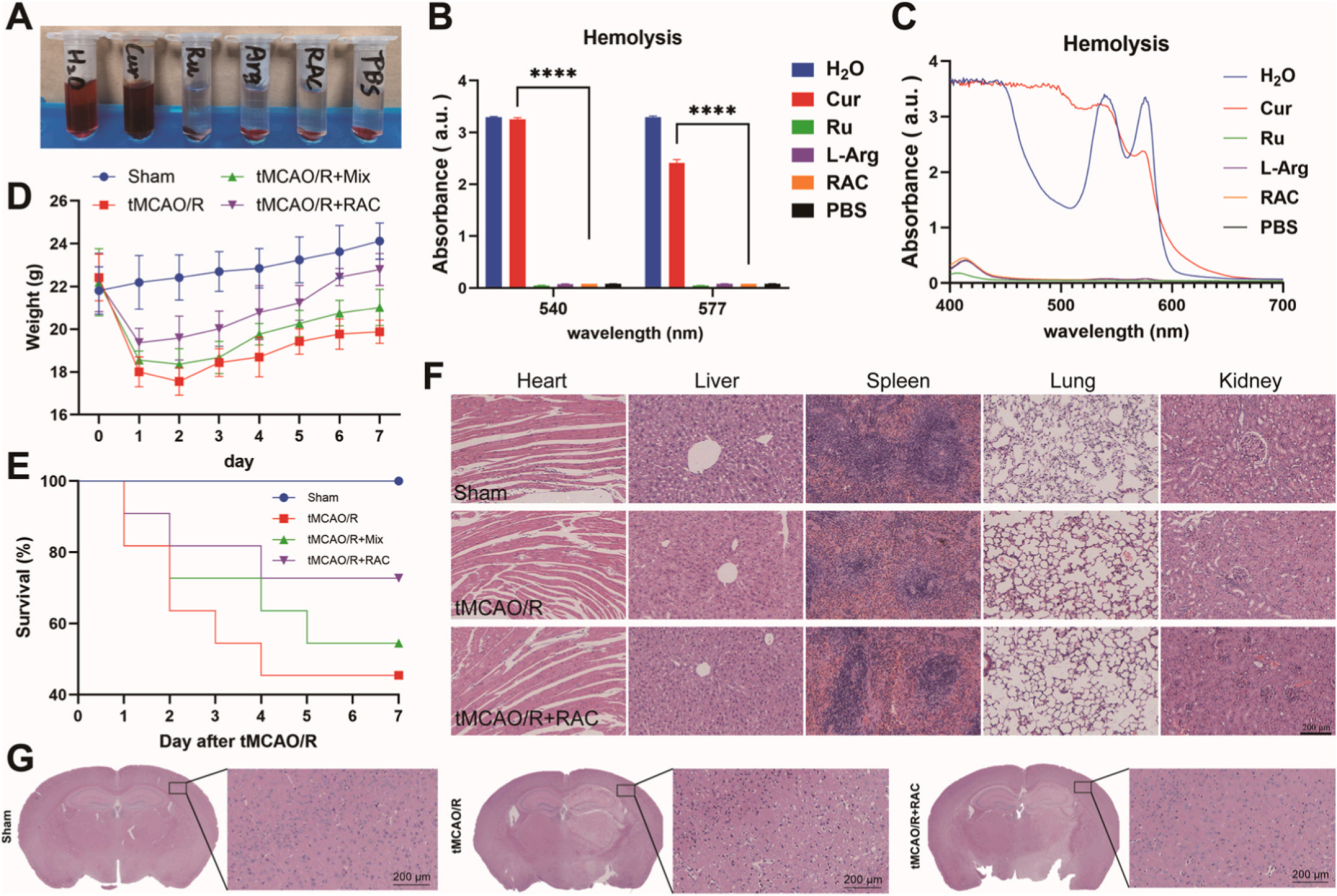
Biocompatibility, *in vivo* tolerability, and histopathological evaluation of RAC. (A) Visual inspection of hemolysis in mouse erythrocytes after incubation with distilled water, PBS, Cur, Ru, L-Arg, or RAC. (B, C) Quantitative evaluation of hemocompatibility. (D) Body weight changes during the observation period. (E) Kaplan-Meier survival curves of mice after different treatments. (F) Representative H&E staining images of major organs, including the heart, liver, spleen, lung, and kidney. (G) Representative H&E staining images of brain tissues at 24 h post-reperfusion. Data are presented as mean ± SEM unless otherwise indicated. Survival curves were compared using the log-rank test. Statistical significance is indicated by ****P < 0.0001.

Hemolysis represents a critical safety concern for systemically administered nanomaterials [47]. Standardized *in vitro* hemolysis assays were performed using mouse erythrocytes exposed to individual components or the integrated RAC at the tested concentrations. Visual inspection revealed striking differences in the supernatant transparency (Fig. 6A). Distilled water and free Cur induced extensive hemolysis, as evidenced by transparent red supernatants, RAC treatment maintained intact erythrocytes with clear supernatants and dense red cell pellets, comparable to the PBS negative controls. Spectrophotometric quantification confirmed that RAC remained below the 5% threshold, meeting the commonly accepted requirement for intravenous biomaterials (Fig. 6B and C). These results suggest that RAC improves hemocompatibility compared with free Cur, likely because incorporation of Cur into the Ru/L-Arg/Cur supramolecular nanoassembly reduces its direct interaction with erythrocyte membranes.

#### 2.12.2 *In vivo* tolerability and histopathological evaluation

To further assess the systemic tolerability of RAC under ischemic stroke conditions, dynamic body weight monitoring was performed throughout the post-treatment observation period (Fig. 6D). Mice in the sham group maintained relatively stable body weight, whereas tMCAO/R mice exhibited progressive body weight loss, likely due to neurological deficits and reduced food intake. RAC treatment attenuated body weight loss compared with the model group, suggesting improved overall physiological status after therapy.

Kaplan-Meier survival analysis further demonstrated the favorable *in vivo* tolerability and therapeutic benefit of RAC (Fig. 6E). Compared with the model group, RAC-treated mice showed higher survival rates during the observation period, supporting the conclusion that RAC not only improves neurological outcomes but also contributes to better overall survival under ischemic stroke conditions.

To evaluate systemic histological safety, H&E staining was performed on major peripheral organs, including the heart, liver, spleen, lungs, and kidneys (Fig. 6F). No observable morphological abnormalities, inflammatory infiltration, or tissue necrosis were detected across the examined organs, indicating that RAC did not induce evident acute organ toxicity under the present experimental conditions.

The neuroprotective efficacy of RAC was further corroborated by H&E staining of brain tissues at 24 h post-reperfusion (Fig. 6G). Brain sections from tMCAO/R mice exhibited profound tissue disruption, including neuronal loss, cytoplasmic vacuolation, and interstitial edema. In contrast, RAC treatment preserved cortical architecture and attenuated edematous changes, consistent with the neuroprotective outcomes observed in TTC and Nissl staining.

These findings collectively indicate that RAC exhibits favorable preliminary biocompatibility, *in vivo* tolerability, and neuroprotective histological effects under the present experimental conditions, supporting its potential for further investigation in AIS therapy.

## 3 Conclusions

In summary, this study presents a ROS-responsive carrier-free nanodrug, RAC, assembled through microwave-assisted coordination among Ru, L-Arg, and Cur for multi-targeted AIS therapy. RAC consistently outperformed the corresponding physical mixture in cellular protection, cerebral blood flow restoration, infarct reduction, neuronal preservation, and neurological improvement, demonstrating that coordination-driven nanoassembly enables therapeutic amplification beyond simple component addition. By integrating ROS scavenging, H_2_O_2_-responsive oxygen generation, NO-related nitrite signaling, ROS/acidosis-responsive Cur release, and cerebral perfusion restoration, RAC provides a coordinated therapeutic strategy for ischemic microenvironment-responsive neuroprotection. Although further studies are needed to clarify its long-term biodistribution, clearance, delayed-treatment efficacy, and precise tissue-level localization, the present findings highlight assembly-dependent carrier-free nanodrug design as a promising strategy for multi-targeted acute ischemic stroke therapy.

## Experimental section

Experimental details are provided in the Supporting Information.

## CRediT authorship contribution statement

**Wanquan Lin:** Conceptualization, Data curation, Formal analysis, Investigation, Methodology, Validation, Writing – original draft, Writing – review & editing. **Chenmin Fan:** Data curation, Methodology, Validation, Writing – review & editing. **Guoyu Xia:** Conceptualization, Data curation, Methodology, Validation. **Ruifei Li:** Methodology. **Hongtao Liu:** Funding acquisition, Project administration, Resources, Supervision. **Zhongxiong Fan:** Funding acquisition, Supervision, Writing – review & editing. **Weiling He:** Funding acquisition, Resources, Supervision, Writing – review & editing. **Jingwen Li:** Funding acquisition, Project administration, Supervision, Writing – review & editing.

## Declaration of competing interest

The authors declare the following financial interests/personal relationships which may be considered as potential competing interests: Jingwen Li reports financial support was provided by Natural Science Foundation of Heilongjiang Province (No.LH2024H080). Hongtao Liu reports financial support was provided by Natural Science Foundation of Hebei Province (No.H2022307031). If there are other authors, they declare that they have no known competing financial interests or personal relationships that could have appeared to influence the work reported in this paper.

## Data Availability

Data will be made available on request.
