# Supplementary material for: ROS-responsive carrier-free nanodrugs for three-pronged acute ischemic stroke therapy
Source: Mater Today Bio. 2026 Aug 4;40:103531. doi: 10.1016/j.mtbio.2026.103531 (PMC13475542; doi:10.1016/j.mtbio.2026.103531)
Supplement: Supplementary file 1 [file mmc1.docx]

**Supplementary Material**

**ROS-responsive carrier-free nanodrugs for three-pronged acute ischemic stroke therapy**

Wanquan Lin ^1,4§^, Chenmin Fan ^3§^, Guoyu Xia ^2§^, Ruifei Li ^4^, Hongtao Liu ^5*^, Zhongxiong Fan ^2*^, Weiling He ^1*^, Jingwen Li ^4, 6*^

^1^ Department of Gastrointestinal Surgery, Xiang'an Hospital of Xiamen University, School of Medicine, Xiamen University, Xiamen, Fujian, 361000, China

^2^ School of Pharmaceutical Sciences, Institute of Materia Medica, Xinjiang University, Urumqi 830017, China

^3^ Department of Cardiology, Fujian Medical Center for Cardiovascular Diseases, Fujian Medical University Union Hospital, Fuzhou, Fujian, P.R. China

^4^ Department of Neurosurgery, Xiang'an Hospital of Xiamen University, School of Medicine, Xiamen University, Xiamen, Fujian, 361000, China

^5^ Department of Pharmacy, The First Hospital of Hebei Medical University, Shijiazhuang, 050000, P.R. China

^6^ The First Hospital of Qiqihar, No. 700 South Pukui Road, Qiqihar, Heilongjiang Province 161000, China.

* Corresponding author

Jingwen Li: ljw010399@163.com, Weiling He: wlhe@xah.xmu.edu.cn,

Zhongxiong Fan: [fanzhongxiong@xju.edu.cn](mailto:fanzhongxiong@xju.edu.cn), Hongtao Liu: lhtyl16@126.com

^§^W.L., C.F., and G.X. contributed equally to this work

1. **Material and methods**

**1.1. Materials**

Ruthenium (III) chloride hydrate (RuCl3·xH2O) and L-arginine (L-Arg) were acquired from Shanghai Yuanye Biotechnology Co., Ltd. (Shanghai, China). Curcumin (Cur) was obtained from Shanghai Macklin Biochemical Co., Ltd. (Shanghai, China). The Cell Counting Kit-8 (CCK-8) assay kit was purchased from Beyotime Biotechnology Co., Ltd. (Shanghai, China). Unless otherwise noted, reagents were of analytical grade, sourced commercially, and utilized without additional refinement. Cell culture reagents, including phosphate-buffered saline (PBS), fetal bovine serum (FBS), Dulbecco's modified Eagle's medium (DMEM), and trypsin-EDTA solution, were supplied by HyClone (Logan, UT, USA). N2a neuronal cells were procured from the National Biomedical Experimental Cell Resource Bank (Beijing, China). For *in vivo* studies, adult male C57BL/6J mice (8 weeks old, 20-25 g) were sourced from Beijing Vital River Laboratory Animal Corporation Limited (Beijing, China).

**1.2. Cell culture**

N2a neuronal cells were cultured in DMEM supplemented with 10% FBS and 1% penicillin-streptomycin at 37 °C in a humidified incubator with 5% CO_2_.

**1.3. Animals**

Adult male C57BL/6J mice (8 weeks old, 20-25 g) were housed in groups of three to four per cage under a 12 h light/dark cycle at 25 °C with ad libitum access to food and water. The animal study was approved by the Animal Care and Use Committee of Xiamen University (Approval No. XMULAC20240023). The study adhered to the committee’s guidelines and fully complied with the Guide for the Care and Use of Laboratory Animals published by the National Institutes of Health. Efforts were made to minimize animal suffering and to reduce the number of animals used.

**1.4.** **Synthesis of RAC**

RAC was prepared by a microwave-assisted coordination-driven self-assembly. Briefly, RuCl3·xH2O (9 mg), L-Arg (6 mg), and Cur (3 mg) were mixed at a mass ratio of 3:2:1 and dissolved in methanol (3 mL). After vigorous stirring for 5 min, the mixture was transferred to a sealed microwave reaction vessel and heated at 100 ℃ for 10 min at 300 W. After cooling to room temperature, excess diethyl ether was added to precipitate the product. The precipitate was collected by centrifugation at 8000 rpm for 10 min, washed three times with diethyl ether, and lyophilized to obtain RAC as a dark brown powder. The product was stored at -20 °C under an inert atmosphere until use. The physical mixture (Mix) was prepared by simply mixing RuCl₃·xH₂O, L-Arg, and Cur at the same mass ratio of 3:2:1 without microwave irradiation.

**1.5. Characterization of RAC**

The hydrodynamic size and zeta potential of RAC were measured by dynamic light scattering and electrophoretic light scattering using a Zetasizer Nano ZS (Malvern Instruments, UK). Surface morphology and elemental distribution were characterized by scanning electron microscopy equipped with energy-dispersive X-ray spectroscopy (Helios 5 UC, Thermo Fisher Scientific, USA). The internal structure was examined by transmission electron microscopy (Talos F200, FEI, USA) at 200 kV, and crystallinity was assessed by selected-area electron diffraction (SAED). Fourier transform infrared spectra were collected on a Nicolet iS10 spectrometer (Thermo Fisher Scientific, USA) in attenuated total reflection mode. Powder X-ray diffraction patterns were recorded on a D8-A25 diffractometer (Bruker, Germany) using Cu Kα radiation. X-ray photoelectron spectroscopy measurements were performed on an Escalab Xi+ system (Thermo Fisher Scientific, USA). UV-Vis spectra were acquired on a UV-2550 spectrophotometer (Shimadzu, Japan).

**1.6. *In vitro* on-demand drug release**

Cur release from RAC was evaluated using a dialysis method under simulated physiological and ischemic conditions. RAC (3 mg) was dispersed in PBS (3 mL) with or without H₂O₂ (100 or 200 μmol/L). The dispersion was loaded into dialysis tubing (molecular weight cut-off: 500 Da) and immersed in the corresponding release medium (47 mL) at pH 7.4, pH 6.5, or pH 5.0. The system was maintained at 37 °C with shaking at 100 rpm. At predetermined time points (0, 0.5, 2, 4, 6, and 8 h), 3 mL of the release medium was withdrawn and replaced with an equal volume of fresh medium. Cur concentration was determined by UV–Vis spectroscopy at its characteristic wavelength, and Cur release was calculated accordingly.

$$\text{C}\text{umulative}\text{ }\text{R}\text{el}\text{ease}\text{ (\%) = }\frac{\text{3}\text{× }\text{∑}_{\text{i}\text{=1}}^{\text{n}\text{−1}}\text{C}\text{i}\text{+}\text{50}\text{×}\text{C}\text{n}}{\text{weight}\text{ }\text{of}\text{ }\text{drug}\text{ }\text{in}\text{ }\text{nanoreactors}\text{ }}\text{ }\text{*}\text{ }\text{100\%}$$

**1.7. *In vitro* cytotoxicity**

N2a cells were seeded in 96-well plates at a density of 3 × 104 cells per well and incubated overnight. Cells were then treated with RuCl3, L-Arg, Cur, or RAC at serial concentrations for 24 h. For time-dependent cytotoxicity of RAC, cells were incubated for 4, 8, 12, and 24 h. After treatment, cells were washed once with PBS and incubated with fresh medium containing 10 % CCK-8 reagent (100 μL per well) for 1 h at 37 °C in the dark. Absorbance at 450 nm was measured using a microplate reader (Multiskan GO 1510, Thermo Fisher Scientific).

**1.8. Oxygen-glucose deprivation/reperfusion (OGD/R) model**

N2a cells were subjected to OGD/R to mimic ischemia–reperfusion injury *in vitro*. Cells were washed three times with PBS and incubated with pre-warmed glucose-free DMEM (serum- and antibiotic-free). Plates were placed in a hypoxia chamber (95 % N_2_, 5 % CO_2_) at 37 °C for 2 h. For reperfusion, the medium was replaced with complete DMEM, and cells were returned to a normoxic incubator (37 °C, 5 % CO_2_) for 24 h.

**1.9. *In vitro* specific cellular uptake**

To quantify cellular uptake of Cur or RAC in N2a cells, flow cytometry was performed by taking advantage of the intrinsic fluorescence of Cur. N2a cells were seeded in 12-well plates at a density of 3×10^4^ cells per well and cultured overnight. Cells were then incubated with free Cur or RAC for 2, 4, 6, and 8 h. At each time point, cells were harvested by scraping from 12-well microplates and collected by centrifugation for flow cytometric analysis.

**1.10. Flow cytometry assay**

N2a cells were obtained by scraping from 12-well microplates, and extracted by centrifuging and filtered for flow cytometry. A Beckman CytoFLEX flow cytometer was used for signal acquisition. Singlet cell populations were identified for quantitative comparison among groups.

**1.11. Apoptosis detection by flow cytometry**

Cellular apoptosis was quantified using the Annexin V/PI apoptosis detection kit (Beyotime). For Nrf2 inhibition, cells in the indicated groups were pretreated with ML385 (5 μM) for 2 h before RAC treatment under OGD/R conditions. After treatment, N2a cells were harvested, washed twice with cold PBS, and resuspended in binding buffer. Cells were stained with Annexin V and PI for 15 min at room temperature in the dark, then analyzed by flow cytometry (Beckman CytoFLEX). Early apoptosis (Annexin V⁺/PI⁻) and late apoptosis (Annexin V⁺/PI⁺) were quantified by flow cytometry.

**1.12. Mitochondrial membrane potential (JC-1) assay**

Mitochondrial membrane potential was evaluated using the JC-1 assay kit (Beyotime). After OGD/R treatment, N2a cells were incubated with JC-1 working solution for 20 min at 37 °C. In healthy cells, JC-1 forms aggregates emitting red fluorescence (excitation 525 nm / emission 590 nm), while in depolarized mitochondria, JC-1 exists as monomers emitting green fluorescence (excitation 488 nm / emission 529 nm). JC-1 red and green fluorescence distributions/percentages were quantified by flow cytometry and visualized by CLSM.

**1.13. Intracellular ROS detection by DCFH-DA and DHE staining**

Intracellular ROS levels were assessed using 2′,7′-dichlorofluorescein diacetate (DCFH-DA) and dihydroethidium (DHE) fluorescent probes. For DCFH-DA staining, N2a cells were incubated with DCFH-DA (10 μmol/L) for 30 min at 37 °C after drug treatment under OGD/R conditions. For DHE staining, cells were incubated with DHE (5 μmol/L) for 30 min at 37 °C. Fluorescence intensity was quantified by flow cytometry (Beckman CytoFLEX) and visualized by confocal laser scanning microscopy.

**1.14. *In vitro* BBB evaluation**

bEnd.3 cells were used to establish the *in vitro* BBB model. In a typical procedure, bEnd.3 cells were seeded in the upper chambers (Corning, USA) with a density of 1.5 × 10^5^ cells per well and then incubated for 7 d until the transendothelial electrical resistance (TEER) reached 200 Ώ·cm^2^. After that, N2a cells were cultured in the lower chambers with a density of 1 × 10^5^ cells per well. After cell adherence, free Cur and RAC were added to the upper chamber at the same Cur-equivalent concentration after incubation for 8 h. Next, the internalization of Cur or RAC in N2a cells was evaluated by CLSM.

**1.15. Griess assay for nitrite detection**

Nitrite production, as an indicator of NO generation, was measured using the Griess assay. RAC solutions were added to 96-well plates at a volume of 50 μL per well, with final concentrations of 10, 20, 40, and 80 μg/mL, followed by 50 μL of 30% H₂O₂. Subsequently, 50 μL each of Griess Reagent I and II were added sequentially. The absorbance at 540 nm was measured using a microplate reader after incubation for 1, 15, 30, 60, 120, 240, 360, and 720 min. Nitrite concentrations were determined using a sodium nitrite standard curve to evaluate the time- and concentration-dependent NO-generating properties of RAC.

**1.16. Laser speckle contrast imaging (LSCI)**

Cerebral cortical perfusion was assessed by laser speckle contrast imaging at 24 h post-reperfusion. Mice were anesthetized with 1.5% isoflurane and placed in a stereotactic frame. The skull was exposed by midline incision, and a laser speckle perfusion imager (PeriCam PSI, Perimed) was used to acquire two-dimensional blood flow maps of the cortical surface. The reduction in relative cerebral blood flow (rCBF) was calculated as [1 − (CBF in the ischemic hemisphere / CBF in the contralateral hemisphere)] × 100%. In addition to the model and RAC groups, single-component controls (Ru, L-Arg, Cur), and the physical mixture (Mix) were included. An additional tMCAO/R+RAC+cPTIO group was included in the LSCI experiment to evaluate the contribution of NO-related signaling to RAC-mediated cerebral perfusion improvement. cPTIO (2-(4-carboxyphenyl)-4,4,5,5-tetramethylimidazoline-1-oxyl-3-oxide) was freshly prepared in sterile normal saline and administered intraperitoneally (i.p.) at 0.6 mg/kg 2 h before tMCAO/R induction.

**1.17. Hemolysis trial**

Hemocompatibility was evaluated by a standard hemolysis assay using fresh mouse erythrocytes. Whole blood was collected from healthy C57BL/6J mice into anticoagulant-containing tubes. Erythrocytes were isolated by centrifugation at 1500 rpm for 10 min at 4 °C and washed three times with PBS. The red blood cells (RBCs) were then resuspended in PBS to obtain a 2.5 % (v/v) RBC suspension. Aliquots (200 μL) of RBC suspension were mixed with 0.5 mL of deionized water (positive control), PBS (negative control), Cur (0.5 mg/mL), RuCl_3_ (0.5 mg/mL), L-Arg (0.5 mg/mL), or RAC (0.5 mg/mL). After incubation at 37 °C for 2 h with gentle shaking, samples were centrifuged at 2500 rpm for 10 min at 4 °C. The supernatants were transferred to a 96-well plate and the absorbance was measured at 540 nm and 577 nm using a microplate reader (Synergy H1, BioTek).

**1.18. Transient middle cerebral artery occlusion followed by reperfusion (tMCAO/R) model**

tMCAO/R was established in adult male C57BL/6J mice. Mice were anesthetized by intraperitoneal injection of 1.25 % tribromoethanol (200 μL per 10 g body weight). A 6-0 nylon monofilament with a silicone-coated tip was introduced into the right internal carotid artery via the external carotid artery and advanced approximately 9-10 mm beyond the carotid bifurcation to occlude the origin of the middle cerebral artery. After 60 min of occlusion, the filament was withdrawn to allow reperfusion. Sham-operated mice underwent the same procedure without filament insertion. Body temperature was maintained at 37 °C using a thermostatically controlled heating pad. Animals exhibiting subarachnoid hemorrhage were excluded from subsequent analyses. For drug treatment, RAC or Mix was administered immediately after filament withdrawal by intravenous (tail vein) injection at a dose of 10 mg/kg.

**1.19. *In vivo* brain-associated exposure of RAC**

To evaluate brain-associated exposure *in vivo*, ex vivo brain fluorescence imaging was performed by utilizing Cur as an intrinsic fluorescent tracer. C57BL/6J mice were randomly assigned to two groups: free Cur and RAC. Mice received a single tail-vein injection (200 μL) of the corresponding formulation at an equivalent Cur dose. At 12 h post-injection, mice were euthanized under anesthesia, and brains were harvested and rinsed with PBS. Fluorescence images of the brains were acquired using an IVIS Lumina III imaging system (PerkinElmer, USA) under identical acquisition settings. Quantification of radiant efficiency in the brain was performed using Living Image software (v4.7.3) for semi-quantitative comparison between groups.

**1.20. 2,3,5-triphenyltetrazolium chloride (TTC) staining**

At 24 h post-reperfusion, mice were euthanized and brains were rapidly removed. Brains were briefly placed at -20 °C for 15 min and sectioned coronally into 2.5 mm slices using a brain matrix. Slices were incubated in 2 % TTC solution (37 °C) for 30 min in the dark, then fixed in 4 % paraformaldehyde at 4 °C for 12 h. Images were acquired, and infarct areas were quantified using ImageJ.

**1.21. Nissl body staining**

Frozen brain sections were air-dried for 1.5 h and rehydrated through graded ethanol. Sections were stained with 1 percent toluidine blue for 20 min, briefly rinsed with distilled water, and differentiated in 70 % ethanol for 5-15 s. After dehydration through graded ethanol and clearing in xylene, sections were mounted with a neutral mounting medium.

**1.22. Neurological severity score**

Neurological function was evaluated at 24 h post-reperfusion using the Bederson and Clark scoring systems by investigators blinded to treatment. For Bederson scoring, mice were graded as 0 (no deficit), 1 (forelimb flexion), 2 (consistent circling), 3 (falling to the contralateral side), or 4 (no spontaneous walking with depressed consciousness). For Clark scoring, neurological performance was evaluated across gait, circling behavior, climbing, posture symmetry, induced circling, whisker response, and forelimb outstretching, with each item scored from 0 (normal) to 4 (severe impairment); the sum of all items was used as the final score.

**1.23. Hematoxylin and eosin (H&E) staining**

Frozen brain sections were air-dried for 90 min, stained with hematoxylin for 8 min, briefly differentiated in 1 percent HCl-ethanol for 1-2 s, and counterstained with eosin for 4 min. Sections were dehydrated through graded ethanol, cleared in xylene, and mounted with a neutral mounting medium.

**1.24. Statistical analysis**

All data are presented as mean ± SEM unless otherwise indicated. Statistical analyses were performed using unpaired two-tailed Student's t-test (two groups) or one-way analysis of variance (ANOVA) followed by Tukey's post hoc test (multiple groups). Survival curves were compared using the log-rank test when applicable. Statistical significance was defined as P < 0.05. Significance levels are denoted as * P < 0.05, ** P < 0.01, *** P < 0.001, and ****P < 0.0001.

2. **Supporting Data**

Figure S1. Change in Size of RAC in DMEM supplemented with 10% fetal bovine serum for 7 d.

Figure S2. SAED pattern of RAC, showing diffuse rings characteristic of an amorphous or poorly crystalline structure.

Figure S3. UV–Vis calibration curve of Cur used for quantification in the *in vitro* release study.

Figure S4. Photographs of methylene blue (MB) solutions in the hydroxyl-radical generating Fenton system in the presence of RuCl3, L-Arg, Cur, or RAC. The control group was prepared without Fenton reagents.

Figure S5. *In vitro* cytotoxicity of individual components and RAC in N2a cells. (A) Cell viability after 24 h incubation with RuCl3, L-Arg, Cur, or RAC at the indicated concentrations. (B) Time-dependent viability of N2a cells treated with RAC for 4-24 h. Data are presented as the means ± SEM. (n = 4).

Figure S6. Cellular uptake of free Cur and RAC in N2a cells. (A) Representative flow cytometric histograms showing time-dependent uptake of free Cur and RAC at 2, 4, 6, and 8 h. (B) Quantitative analysis of mean fluorescence intensity (MFI) at the indicated time points. Data are presented as the means ± SEM. (n = 3). **P < 0.01, ****P < 0.0001.

Figure S7. Dose optimization of RAC for protecting N2a neuronal cells against OGD/R injury. N2a cell viability was measured by CCK-8 and normalized to the normoxia group. Data are presented as the means ± SEM. (n = 6). ****P < 0.0001.
